# Roles of Macrophages in the Development and Treatment of Gut Inflammation

**DOI:** 10.3389/fcell.2021.625423

**Published:** 2021-03-02

**Authors:** Xuebing Han, Sujuan Ding, Hongmei Jiang, Gang Liu

**Affiliations:** College of Bioscience and Biotechnology, Hunan Agricultural University, Hunan Provincial Engineering Research Center of Applied Microbial Resources Development for Livestock and Poultry, Changsha, China

**Keywords:** macrophages, inflammation, homeostasis, intestinal, treatment

## Abstract

Macrophages, which are functional plasticity cells, have the ability to phagocytize and digest foreign substances and acquire pro-(M1-like) or anti-inflammatory (M2-like) phenotypes according to their microenvironment. The large number of macrophages in the intestinal tract, play a significant role in maintaining the homeostasis of microorganisms on the surface of the intestinal mucosa and in the continuous renewal of intestinal epithelial cells. They are not only responsible for innate immunity, but also participate in the development of intestinal inflammation. A clear understanding of the function of macrophages, as well as their role in pathogens and inflammatory response, will delineate the next steps in the treatment of intestinal inflammatory diseases. In this review, we discuss the origin and development of macrophages and their role in the intestinal inflammatory response or infection. In addition, the effects of macrophages in the occurrence and development of inflammatory bowel disease (IBD), and their role in inducing fibrosis, activating T cells, reducing colitis, and treating intestinal inflammation were also reviewed in this paper.

## Introduction

The intestinal tract is the largest independent immune system in the body. It is continuously exposed to foreign antigens and the distinction between harmful and harmless antigens is necessary for the intestine to ensure an appropriate response to every antigen (Weiner, 2000; Ma et al., 2020a). The gut needs to produce a strong protective immune response to resist the invasion of pathogenic antigens, while similar reactions to harmless antigens such as dietary proteins or symbiotic microorganisms, may lead to chronic inflammatory diseases. Macrophages are phagocytes found in tissues and maintain tissue homeostasis, regulate inflammation, and play a significant role in host protection. There are many microorganisms colonized in the human intestine, and more than 1000 bacterial species in the intestinal ecosystem of a single individual. Among them, Actinobacteria, Bacteroidetes, Firmicutes, Proteobacteria, and Tenericutes are the predominant bacterial phyla, while the abundances of Fusobacteria, Saccharibacteria, Spirochaetes, Synergistetes and Verrucomicrobia are lower (Fassarella et al., 2020).

The production of phagocytic cytotoxic substances by activated macrophages is a key process in the control of intracellular pathogens (Piacenza et al., 2019). The pattern recognition receptors on the surface of macrophages recognize and bind to the corresponding pathogen associated molecular pattern (PAMP)—a specific molecular structure shared by some pathogens—on pathogens, and nonspecifically phagocytize and remove pathogenic microorganisms (Ley et al., 2016). Different kinds of microorganisms express different PAMPs, including mainly lipopolysaccharides (LPSs), phosphoteichoic acid, peptidoglycan, and other structures that usually do not exist in the host. Then, the pathogens are phagocytized and digested by macrophages, and the lymphocytes or other immune cells are activated to kill these pathogens (Jain et al., 2019). On the other hand, phagosomes are formed when the pathogen is engulfed by macrophages and fuse with lysosomes to release enzymes and toxic substances, resulting in killing or having cytotoxic effects on bacteria and tumor cells. The intestinal mucosa is the first line of defense for organisms against intestinal pathogens. The lamina propria of the small intestine is the main site of the intestinal immune system, which contains a large number of macrophages, CD4 T cells, and dendritic cells. These cells play a key role in early resistance to intestinal pathogens. Macrophages play a significant role in many processes, such as the human immune function, parasite infection, and tissue remodeling by secreting cytokines and producing reactive oxygen and nitrogen intermediates. In a broad sense, intestinal macrophages are divided into two categories: resident and inflammatory (Mills et al., 2000). The former maintains intestinal health, while the latter plays an important role in the occurrence of inflammatory reactions. Multiple studies have shown that macrophages are associated with the development of intestinal inflammation and secrete a large number of cytokines and bioactive substances that participate in the inflammatory response (Cummings et al., 2016; Joeris et al., 2017). Herein, we review the origin and development of macrophages and their role in intestinal inflammation and treatment.

## Intestinal Inflammation

**FIGURE 1:**
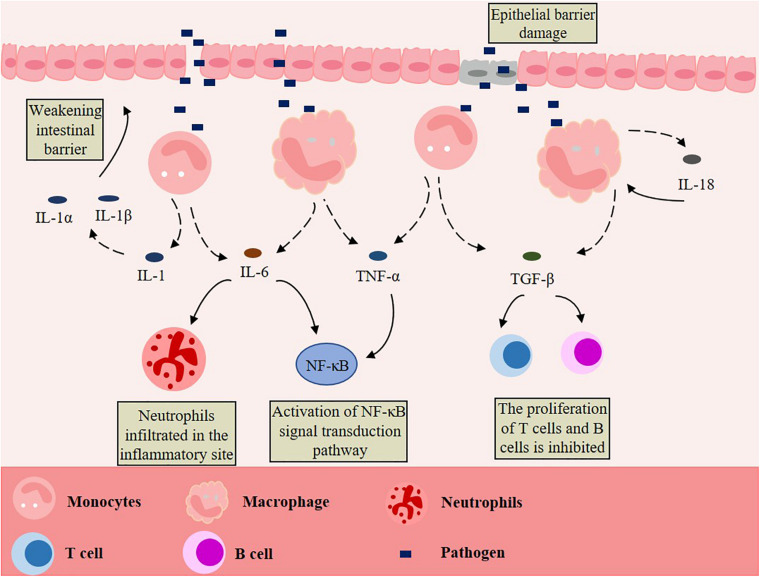
Macrophages promote the development of IBD. Pathogens cross the damaged intestinal epithelial cell barrier and stimulate macrophages to produce pro-inflammatory cytokines, such as interleukin-1 (IL-1), IL-6, IL-18, transforming growth factor-α (TGF-α) and tumor necrosis factor-β (TNF-β) are released. These act on intestinal epithelial cells directly or indirectly, leading to the injury or necrosis of these cells, thus promoting the occurrence and development of IBD.

The healthy gut can control inflammation through its powerful mechanisms, but inflammatory bowel disease (IBD) can occur if the inflammation is not resolved (Murray and Smale, 2012). IBD, which includes Crohn’s disease and ulcerative colitis, is a kind of chronic gastrointestinal inflammatory disease with unknown etiology and recurrent attacks (Torres et al., 2017; Ungaro et al., 2017). The pathogenesis of IBD is unknown, but it is believed that the uncontrolled immune response of genetically predisposed individuals to environmental factors and intestinal microorganisms is the cause (Khor et al., 2011; Kostic et al., 2014; de Souza and Fiocchi, 2016; Liu and Stappenbeck, 2016; Ni et al., 2017; Ananthakrishnan et al., 2018). In other words, the combined effects of genetic, microbial, immune, and environmental factors lead to an abnormal and excessive immune response of the commensal microbiota (Wallace et al., 2014). When the intestine is invaded by pathogens, which can cross the damaged intestinal epithelial cell barrier, the intrinsic defense cells in the epithelium, especially the macrophages, will produce pro-inflammatory cytokines after being stimulated, and then release interleukin-1 (IL-1), IL-6, IL-18, transforming growth factor-β (TGF-β), and tumor necrosis factor-α (TNF-α). These cytokines directly or indirectly affect the intestinal epithelial cells, leading to the injury or necrosis of these cells, which promotes the occurrence and development of IBD (Figure 1). An over-secretion of cytokines and chronic inflammation are the typical features of IBD, with clinical symptoms of diarrhea, abdominal pain, fever, intestinal obstruction, and disability symptoms of blood or mucus or both (Baumgart and Carding, 2007; Geremia et al., 2014; Geremia and Arancibia-Cárcamo, 2017; Hidalgo-Garcia et al., 2018; Ding et al., 2019b). IBD occurs exclusively in the colon in ulcerative colitis and almost anywhere along the gastrointestinal tract in chronic diarrhea (Jones et al., 2018). In addition, IBD also has the characteristics of intestinal microbiota dysbiosis. Compared with the gut of healthy people, the quantity and diversity of intestinal bacteria is lower in IBD patients (Frank et al., 2007; Lepage et al., 2011; Kostic et al., 2014). In some sufferers, the inflammation of the mucosa is associated with these changes and bacterial factors (Gevers et al., 2014; Franzosa et al., 2019; Lloyd-Price et al., 2019). Some scholars have analyzed the pro-inflammatory and anti-inflammatory pathways of IBD patients, and the results show that the imbalance of immune responses is caused by the change of balance among inflammatory, regulatory and anti-inflammatory cytokines (Bouma and Strober, 2003). When IBD occurs, monocyte infiltration will increase and produce many pro-inflammatory mediators, including TNFα, IL-1, IL-23, and nitric oxide (Ogino et al., 2013; Bain and Mowat, 2014; Sanders et al., 2014; Magnusson et al., 2016; Joeris et al., 2017). Many types of mucosal immune cells are related to the pathogenesis of IBD: intestinal epithelial cells, innate arm dendritic cells, innate lymphoid cells, neutrophils, macrophages, Foxp3^+^ regulatory T (Treg) cells of the adaptive arm, interferon-γ-producing type 1 helper T cells (Th1), interferon-γ helper T cells (Th17), and secretory mediators of the adaptive arm of the mucosal immune system—cytokines, chemokines, eicosanoic acid, reactive oxygen species and nitrogen species (Xavier and Podolsky, 2007; Wu et al., 2015). A study has found that, compared with quiescent IBD or the healthy intestine, IBD in active humans was related to the increase of colonic mRNA expression of TNF, IL-1β and IL-6, and of the HLA-DR^*Int*^:HLA-DR^*Hi*^ and CD14^*Hi*^:CD14^*Lo*^ cell ratios (Jones et al., 2018).

Molecular cues are also responsible for the contribution of intestinal macrophages in the development of IBD. Toll-like receptors (TLRs) play a key role in maintaining intestinal homeostasis. After recognizing PAMPs, TCLs are activated to regulate both innate and adaptive immunity. Innate immunity is regulated by mediating the phosphorylation of IκB, thereby activating NF-κB. Moreover, the proliferation and differentiation of Th1 and Th2 from T cells is regulated by TCLs to regulate adaptive immunity. When these regulations are disturbed, the expression of TLRs increases and the downstream signaling cascade is over activated, resulting in the over production of inflammatory cytokines and IBD (Lu et al., 2018). When IBD occurs, it is often accompanied by the death of intestinal epithelial cells (Abraham and Cho, 2009). Epithelial injury and inflammation in IBD patients are usually dependent on TNF (Zeissig et al., 2004). When the production of TNF increased in IBD, the expression of the *TNFAIP3* gene, which encodes A20, also increases. The A20 protein is the negative feedback regulator of NF-κB. In the intestinal epithelium of IBD patients, A20 is expressed by an intestinal epithelial cell specific promoter and is highly sensitive to intestinal epithelial cell death, intestinal injury, and shock induced by TNF (Garcia-Carbonell et al., 2018).

Generally speaking, IBD often occurs in young individuals, and most patients with IBD are expected live to a normal life due to the progress of medical treatment. Despite the low mortality rate of IBD, the incidence rate is still a serious problem. Moreover, IBD is incurable and increases the risk of lymphoma, cholangiocarcinoma, and colorectal cancer (Samadder et al., 2019; Scharl et al., 2019). Many patients with IBD have to undergo surgery multiple times to relieve symptoms, which may lead to postoperative complications and infections, adversely affecting their quality of life (Torres et al., 2017; Ungaro et al., 2017; Liang et al., 2018). There have been some—although relative few—experiments using immunomodulators for IBD treatment, but the effect of the treatment declines with time (Torres et al., 2017; Ungaro et al., 2017; Friedrich et al., 2019). Tissue reparative programs may also contribute to restoring the barrier, but improper regulation may lead to fibrosis and intestinal structuring due to the dysregulation of intestinal, which is a possible complication of IBD (Rieder and Fiocchi, 2009; Rieder et al., 2017).

## Ontogeny, Location, and Characterization of Macrophages

### Origin and Differentiation of Macrophages

**FIGURE 2:**
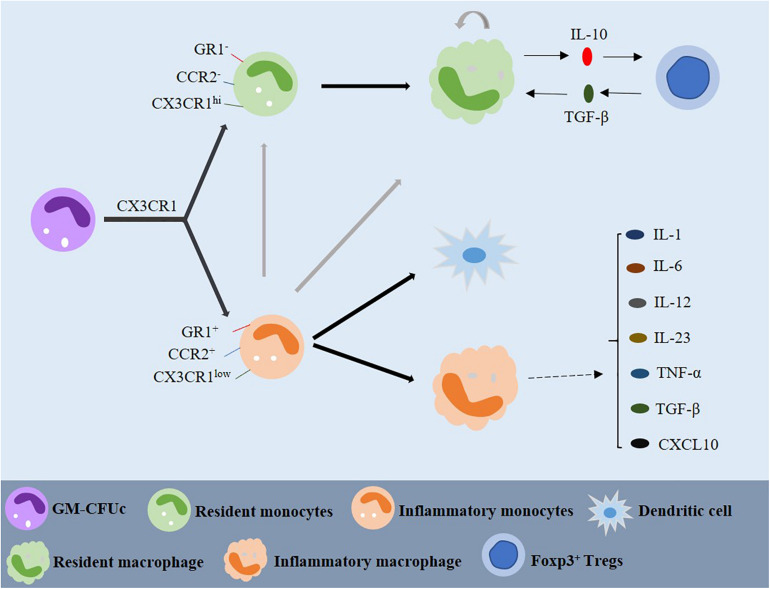
Origin and differentiation of macrophages. GM-CFUc can be divided into resident and inflammatory monocytes by CX3CR1. Inflammatory monocytes may also be one of the sources of resident monocytes. Resident macrophages are usually produced by resident monocytes or, sometimes, by inflammatory monocytes. Resident macrophages and Foxp3^+^ T cells play a significant role in maintaining intestinal homeostasis through IL-10 and TGF-β dependent mechanisms. When there is inflammation in the intestine, inflammatory monocytes migrate to the intestine and differentiate into dendritic cells and inflammatory macrophages, which can produce a variety of cytokines involved in the inflammatory reaction. GM-CFUc: granulocyte macrophage colony forming unit; IL: interleukin; TNF-α: tumor necrosis factor-α; TGF-β: transforming growth factor-β.

Macrophages are white blood cells located in tissues. In general, it is believed that macrophages are derived from monocytes, and monocytes are derived from precursor cells in bone marrow, which is also known as the granulocyte-macrophage colony-forming unit (GM-CFUc) (Figure 2; van Furth and Cohn, 1968; van Furth et al., 1972). However, whether monocytes differentiate into tissue-specific macrophages in the blood is still controversial. Some scholars believe that monocytes continue to develop and mature in the blood, where they can migrate to different tissues to form cell groups with different functions and structures. According to their function during the migration from blood to tissue, they can be divided into “inflammatory” and “resident” monocytes (Geissmann et al., 2003). The resident monocytes are defined as CCR2^–^, CX3CR1^*hi*^, and GR1^–,^ they exist in the non-inflammatory tissues and have a long half-life. The precursor inflammatory monocytes are CCR2^+^, CX3CR1^*low*^, and GR1^+^ are found in the inflammatory tissues, having a short survival time. The two monocyte populations can be distinguished by the expression of CX3CR1, a cell surface marker (Figure 2; Geissmann et al., 2003; Strauss-Ayali et al., 2007). One study has shown that when blood vessels are damaged and infected, the colonized monocytes rapidly invade the tissues, and then initiate the innate immune response and differentiate into macrophages (Figure 2; Geissmann et al., 2003). By contrast, inflammatory monocytes reach the site of inflammatory response and differentiate into inflammatory dendritic cells after infection. It has been demonstrated that inflammatory monocytes can differentiate into inflammatory macrophages, and the migration of resident monocytes may depend on chemical signals from damaged tissues or endothelial cells (Figure 2; Mowat and Bain, 2011).

In the human bone marrow, monocytes can be divided into Ly6C^*lo*^ monocytes and Ly6C^*hi*^ monocytes through the expression of Ly6C/GR1, CCR2, and CX3CR1. Because Ly6C^*hi*^ monocytes tend to perform functions that traditionally belong to monocytes, they are now called “classical” monocytes. On the other hand, some scholars have proposed that Ly6C^*lo*^ monocytes are the precursors of tissue resident macrophages because they do not enter inflammatory tissue (Geissmann et al., 2003). In addition, a study has shown that the “non-classical” Ly6C^*lo*^ monocytes are not used as circulating intermediates, but their main function is to patrol the vascular system and remove necrotic endothelial cells (Carlin et al., 2013). Therefore, Ly6C^*lo*^ monocytes can be considered the macrophages of the circulatory system in some ways. In the normal colon, monocytes gradually differentiate into resident macrophages. When there is inflammation in the gut, resident macrophages still originate from monocytes in blood circulation but change from anti-inflammatory to inflammatory macrophages with high expression of TLRs. However, studies have shown that Ly6C^*hi*^ monocytes can also be converted to Ly6C^*lo*^ monocytes and returned to the bone marrow to replenish the resident macrophages (Figure 2; Gren and Grip, 2016). Some scholars proposed that resident macrophages also have the characteristics of self-renewal (Figure 2; Bain and Mowat, 2011).

Resident macrophages produce anti-inflammatory cytokines, such as IL-10 and TGF-β. Studies have reported that IL-10 produced by macrophages has the effect of regulating the expression of Foxp3^+^ Tregs, and macrophages highly express the TGF-β receptor and participate in the signal transduction of activated TGF-β (Figure 2; Mowat and Bain, 2011; Weinhage et al., 2015). TGF-β can combine with the Foxp3 expressed by Tregs to form CD4^+^ Foxp3^+^ Tregs, which can reduce the activation of macrophages and translocation of NF-κB in the mucosa (Li et al., 2010).

Some studies have shown that many tissue macrophages do not originate from blood mononuclear cells but exist independently of conventional hematopoiesis and originate from embryonic precursors produced by the yolk sac or fetal liver (Schulz et al., 2012; Hashimoto et al., 2013; Yona et al., 2013; Epelman et al., 2014; Sheng et al., 2015). As we know, the differentiation continuum of monocyte to macrophage exists in intestinal lipoprotein, which has been called the monocyte “waterfall” (Tamoutounour et al., 2012; Bain et al., 2013). Ly6C^*hi*^ CX3CR1^*int*^ MHCII^–^ monocytes exist at one end of the “waterfall”, and their phenotype and morphology are similar to those of their counterparts in the blood. In fact, the expression of molecules of monocytes in the mucosa, including CCR2, VLA-1, CD62L, Ly6C and LFA-1, is still preserved; these molecules are related to the chemotaxis and extravasation of circulation (Schridde et al., 2017). First, these monocytes show MHCII expression; Then, the molecules associated with extravasation, including LFA-1, CCR2, and CD62L, are downregulated; Finally, CX3CR1 is upregulated to obtain fully mature macrophages (Tamoutounour et al., 2012; Bain et al., 2013; Schridde et al., 2017). At the same time, it has been proved that the human intestinal mucosa presents a similar “waterfall”, with the classic CD14^*hi*^CCR2^+^CD11C^*hi*^ monocytes and mature CD14^*lo*^CCR2^–^CD11C^*lo*^ macrophages at the two ends (Bain et al., 2013; Bernardo et al., 2018; Bujko et al., 2018).

Inflammation includes the detection of tissue injury or infection, the subsequent inflammatory response and the final resolution. Monocytes are equipped with a large number of scavengers and pattern recognition receptors, which can react to local danger signals quickly. Their high plasticity enables them to adapt to molecular changes in response to the production of effector molecules that drive inflammation. Although we now have a deeper understanding of the function of Ly6C, more research is needed to explain the molecular mechanism of monocytes acting in a restorative rather than pathological manner.

### Distribution of Intestinal Macrophages

Macrophages play a significant role in regulating intestinal peristalsis. They are distributed throughout the gastrointestinal mucosa, with a large proportion of them being located in the natural layer (LP) near the epithelium and a small part of them appear in the smooth muscle layer of the intestinal wall (Tajima et al., 2012; Gabanyi et al., 2016). In different parts of the gastrointestinal tract, the number of macrophages varies in the intestinal mucosa. Both in humans and rodents, the number of macrophages in the colon and lamina propria was found to be more than that in the small intestine (Nagashima et al., 1996; Denning et al., 2011). However, the number of macrophages follows a continuous gradient trend between the proximal and distal intestines of mice, while the number of macrophages in different parts of the colon was similar in mice and humans (Nagashima et al., 1996; Grainger et al., 2017).

### Functional Plasticity of Macrophages

Generally speaking, macrophages are phagocytes in tissues and play an important role in homeostasis of adipose and tissue, regulation of inflammatory response and defense protection of host. Macrophages have the property of plasticity and can change their physiology, being able to produce different cell populations with different functions, according to environmental cues (Mosser and Edwards, 2008). The activation state of macrophages was initially divided into classically activated M1 macrophages and alternatively activated M2 macrophages. Inflammatory macrophages are usually activated as the M1 phenotype, while resident macrophages usually belong to the activated M2 phenotype. M1 and M2 macrophages are induced by interferon-γ (IFN-γ) and IL-4, respectively, and participate in the anti-microbial response and the reaction of wound healing and tissue remodeling, respectively (Stein et al., 1992; Mills et al., 2000). It is difficult to distinguish M1 and M2 *in vivo* due to the mixing of activated M1/M2 macrophages caused by the multitude of stimulations, although the polarization state of prototypes M1/M2 has been established *in vitro* (Martinez and Gordon, 2014). Some studies have shown that macrophages become a continuum of activation states when they are stimulated by certain cytokines or complexes, such as TNF-α, LPS, TGF-β, IL-10, IL-13, Glucocorticoid or the immune complex, and macrophage activation with similar but different transcriptional and functional is subsequently produced along the M1/M2 axis (Martinez and Gordon, 2014; Murray et al., 2014; Xue et al., 2014; Murray, 2017). Moreover, some studies have found that macrophages are activated outside the M1-M2 continuum when they are stimulated by high-density lipoproteins, free fatty acids, or chronic-inflammation-related stimulants (Popov et al., 2008; Xue et al., 2014).

The activation and function of macrophages are complex, but the activated states can be identified and distinguished by the abundance of transcription factors, cytokines, and surface molecules (Table 1). For example, M1 macrophages usually produce high levels of pro-inflammatory cytokines, such as TNF-α, IL-6 and IL-12, and promote the induction of nitric oxide synthase (iNOS) and the expression of indoleamine 2,3-dioxygenase in mice and humans, respectively, while M2 macrophages are generally distinguished by stimuli-specific molecules and more general M2 markers (Murray et al., 2014; Xue et al., 2014). CD206 is one such surface marker induced by IL-4/IL-13 and IL-10 in mice and humans, respectively (Stein et al., 1992; Mantovani et al., 2004; Murray et al., 2014). The expression and activity of arginase I also constitute a marker of M2-polarized macrophages in mice, but not in human (Thomas and Mattila, 2014). The expression of IL-10 in several polarization states of M2 macrophages (except for those induced by IL-4/IL-13) is higher than for M1 macrophages, making it a frequently used marker of M2 macrophages.

**TABLE 1:** Phenotype of macrophages and its correlation with stimulating factors, surface markers, cytokines, and functions.

Phenotype	Origin	Stimuli	Surface makers	Secreted mediator	Functions
M1	Hematopoietic stem cells in bone marrow and progenitor cells in the embryonic yolk sac	IFN-γ, LPS, GM-CSF	MHC-II, CD68, CD80, CD86, CD197, SOCS3, B7	IL-1β,IL-6, IL-10^*low*^, IL-12^*hi*^, IL-18, IL-23, TNF, CXCL9, CXCL10, iNOS	Pro-inflammatory Th1 response, key mediator of several autoimmune diseases
M2		IL-4, IL-13, CSF-1, TGF-β, helminth	CD206, CD200R, CD163, CD86, Arg-1	IL-6, IL-10^*hi*^, IL-12^*low*^, VEGF, CCL17, CCL18, CCL22, IL-13α1, CH13L1	Anti-inflammatory, Th2 activation, wound healing
Mregs		IgG, PG, IL-10, apoptotic cells, GPCRs, adenosine	CD80^*low/int*^, CD86^+^, CD163^*low*^, CD206^*low*^	IL-10^*hi*^, IL-12^*low*^, TGF-β	The regulatory agencies controlling immune response, a bridge between innate immunity and adaptive immunity
TAMs		TME	CD81, CD163, CD206, VCAM-1, MHC-II	IL-6, IL-10, TGF-β, CCL2, CCL17, VEDF, CTSC	Associated with tumors

In addition, macrophages can also differentiate into Mregs and TAM, which have different stimulating factors, surface markers, cytokines and functions (Table 1) (Mosser and Edwards, 2008). However, it is not clear what causes the change of activation status of macrophages, the reasons may be the recruitment of monocytes and their response to local changes, the repolarization between M1 and M2 macrophages, or a combination of the two (Italiani and Boraschi, 2014). The traditional macrophage polarization model is not sufficient to describe the full range of macrophage activity. Due to the increased heterogeneity of macrophages in the gut, further work is needed to analyze the role of macrophage subsets in health and disease. Many technologies have been used to study the heterogeneity of macrophages. For example, single-cell RNA sequencing has been used for the transcriptomic profiling of haematopoietic cells in humans, and macrophage heterogeneity across multiple anatomical sites was mapped, with diverse subsets being identified (Bian et al., 2020). A rapid three-dimensional (3D) printing method was also used in the research of cell heterogeneity. Tang et al. reported a controllable, repeatable, and quantifiable 3D bioprinting model of the glioblastoma microenvironment, simulating the high cell heterogeneity and cell interaction in the tumor microenvironment (Tang et al., 2020). In addition, macrophages have highly specialized functions in different tissues, and their receptors are also different. They may cooperate or compete for ligand recognition, which will affect their function.

## Macrophages in the Intestinal Mucosa During Inflammation

### Intestinal Homeostasis and Its Disruption During Inflammation

The gut, which is exposed to pathogens, commensal microbiota, and food antigens, is one of the main interfaces for contact with the outside ambient. The balance between immune responses to pathogens and tolerance is necessary for this bodily niche in order to maintain intestinal homeostasis and body health (Hill and Artis, 2010; Pabst and Mowat, 2012; Belkaid and Hand, 2014). The intestinal epithelium, which is mainly made up of a single layer of intestinal cells, is tightly connected with adjacent cells to form a critical continuous physical barrier, which regulates the selective permeability of luminal content (Odenwald and Turner, 2017; Chelakkot et al., 2018). Except for those in physical barriers, several other types of epithelial cells produced by stem cells, which are located at the base of the intestinal crypt, also play a role in the homeostasis of the body (Clevers and Bevins, 2013; Peterson and Artis, 2014; Johansson and Hansson, 2016; Martens et al., 2018). There is only one mucus layer in the small intestine, while both an internal and outer layer can be found in the colon, making it a habitat for many microbes (Johansson and Hansson, 2016). After passing through the epithelial barrier, the luminal antigens come in contact with the immune cells in the second and third lymphoid organs in the lamina propria (Buettner and Lochner, 2016; Ahluwalia et al., 2017; Da Silva et al., 2017; Mowat, 2018; Tordesillas and Berin, 2018). After the internalization of mononuclear phagocytes, the treated antigens are presented to lymphocytes to induce oral tolerance and interact with the intestinal flora and dietary factors (Hadis et al., 2011; Pabst and Mowat, 2012; Muller et al., 2014; Chinthrajah et al., 2016; Esterházy et al., 2016; Loschko et al., 2016; Nutsch et al., 2016; Belkaid and Harrison, 2017; Kim et al., 2018; Mowat, 2018). Moreover, conventional dendritic cells can polarize naïve T cells by migration, while macrophages lack the characteristics of active migration, but help to amplify the T cell response of lymphocytes (Gaudino and Kumar, 2019). In addition, intestinal macrophages maintain T cell function by scavenging apoptotic/dead cells, secreting cytokines, and remodeling epithelial cells, thus maintaining tissue homeostasis (Zigmond et al., 2012; Ortega-Gómez et al., 2013; Cerovic et al., 2014; Zigmond et al., 2014; Schett and Neurath, 2018; Sugimoto et al., 2019). These processes of active regulation, and T cell deletion and anergy are associated with maintaining oral tolerance (Sun et al., 2015; Luu et al., 2017; Wawrzyniak et al., 2017; Mowat, 2018). In addition, as a response to microbial induction, conventional dendritic cells also support the conversion of immunoglobulin M and immunoglobulin G to immunoglobulin A on B cells, which is essential for the homeostasis of the intestinal environment because immunoglobulin A inhibits the interaction between microorganisms and epithelial cells by transporting across the epithelial cell layer (Litinskiy et al., 2002; Macpherson et al., 2018; Castro-Dopico and Clatworthy, 2019).

In general, mononuclear phagocytes control the stability of the intestinal environment and the ability to trigger the immune response to pathogens by maintaining immune tolerance to commensal animals and diet (Hadis et al., 2011; Bain and Mowat, 2014; Cerovic et al., 2014; Kim et al., 2018; Leonardi et al., 2018). Ideally, these immune responses can promote inflammation remission and rapid homeostasis recovery in tissues. However, due to the repeated and abnormal activation of the immune system, the chronic inflammatory microenvironment of IBD will be produced in the body (Caër and Wick, 2020). Destruction of intestinal homeostasis, including an immune response to commensal bacteria, dysfunction of the epithelial barrier function, the reduction of nutrient absorption, and changes in tissue autophagy and oxygenation, can induce the recruitment of immune cells (Maloy and Powrie, 2011; Johansson et al., 2013; Peterson and Artis, 2014; Colgan et al., 2016; Ramakrishnan and Shah, 2016; Odenwald and Turner, 2017; Okumura and Takeda, 2017; Ahluwalia et al., 2018; Mowat, 2018; VanDussen et al., 2018). These intestinal defects are associated with IBD, and the gene expression related to the prognosis variation of Crohn’s disease can be detected in mononuclear phagocytes. Thus, we can speculate that mononuclear phagocytes play a significant role in the cellular signaling pathway that regulates tolerance and chronic inflammation in the intestine (Lee et al., 2017).

### The Role of Macrophages in Intestinal Inflammation

The largest macrophage population in the body exists in the gastrointestinal mucosa, which plays a key role in maintaining epithelial and immune homeostasis (Lee et al., 1985; Pull et al., 2005; Isidro and Appleyard, 2016; Guan et al., 2019). When intestinal homeostasis is disturbed, the composition of the intestinal macrophage pool will change greatly. The inflammatory macrophages will accumulate in the intestinal mucosa of patients with Crohn’s disease and ulcerative colitis, for example. Compared with CD14^*low*^, these inflammatory macrophages can be identified by the expression of CD14^*hi*^, which produces multiple inflammatory mediators, such as TNF-α, IL-1, IL-6, ROS mediators, and nitric oxide, which makes them different from macrophages in healthy intestines (Thiesen et al., 2014).

**FIGURE 3:**
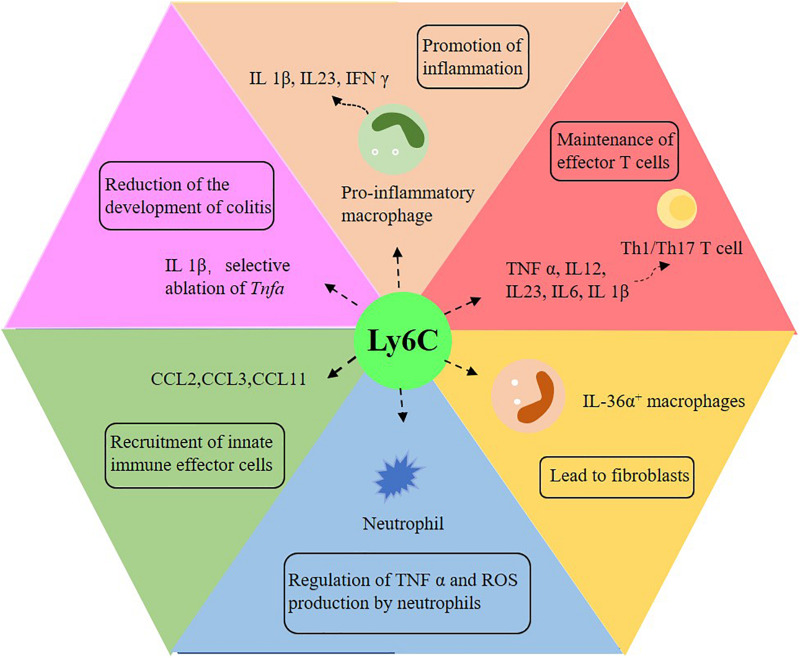
The role of Ly6C monocytes in intestinal inflammation. Ly6C plays an important role in promoting intestinal inflammation, reducing colitis, activating T cells, promoting tissue fibrosis, regulating neutrophils and recruiting innate immune effector cells. IL: interleukin; TNF-α: tumor necrosis factor-α; IFN-γ: interferon-γ; Th1: interferon-γ-producing type 1 helper T cells; Th17: interferon-γ-producing helper T cells.

Ly6C^*hi*^ monocytes and their derivatives play a significant role in intestinal pathology (Bain and Schridde, 2018). When inflammation occurs in the gut, classical monocytes (Ly6C^*hi*^) respond to the stimulation of Toll-like receptors in a highly pro-inflammatory manner, expressing reactive oxygen intermediates (Figure 3; Varol et al., 2009; Weber et al., 2011; Rivollier et al., 2012; Tamoutounour et al., 2012; Zigmond et al., 2012; Bain et al., 2013). CD11c^*high*^CCR2^+^CX3CR1^+^ monocytes infiltrate in the colonic mucosa of IBD patients in a CCR2-dependent manner and cannot completely differentiate into macrophages and produce pro-inflammatory cytokines (Bernardo et al., 2018). Intestinal macrophages in IBD patients produce more pro-inflammatory cytokines, which promote or perpetuate the pathological environment (Kamada et al., 2008; Kamada et al., 2009; Lissner et al., 2015; Barman et al., 2016; Friedrich et al., 2019). In patients with Crohn’s diseases, some factors, such as IFN-γ, induce the differentiation of inflammatory monocytes and the secretion of IL-23, thus creating a vicious circle of inflammation (Kamada et al., 2008). In addition, other mechanisms and disease-related changes in the function of macrophages may also promote the occurrence and development of IBD. For instance, TREM-1^+^ macrophages, which are mainly immature macrophages, increase in frequency and number in patients with IBD, especially in the active lesion area (Schenk et al., 2007; Brynjolfsson et al., 2016). It has also been suggested that bacterial clearance of intestinal macrophages in patients with IBD is impaired, and patients with Crohn’s disease phenotype mainly through dysfunctional autophagy (Smith et al., 2009; Schwerd et al., 2017). After the removal of infectious or inflammatory factors, the intestinal tract must be restored to balance so that a chronic inflammatory reaction will not follow. At the same time, the macrophage pool changes significantly. During colitis in mice, the expansion rate of CX3CR1^*int*^ macrophages returned to normal (Zigmond et al., 2012). On the other hand, Ly6C^*hi*^ monocytes supplement CX3CR1^*hi*^ macrophages in intestinal homeostasis. Once the inflammatory response begins to subside, some of the induced Ly6C^*hi*^ cells may be transformed into resident macrophages with anti-inflammatory effects and may play an active role in tissue injury. IL-1β is believed to be induced mainly by monocytes, and its susceptibility to chemically induced colitis is reduced due to its neutralization (Seo et al., 2015). Meanwhile, the selective ablation of *Tnfa* in Ly6C^*hi*^ monocytes also reduces the development of colitis (Varol et al., 2009). Mice with defective recruitment of inflammatory mucosal monocytes, which is due to the neutralization or deletion of CCL2, CCR2 or β 7 integrins, are protected from colitis induced by DSS (Platt et al., 2010; Takada et al., 2010; Zigmond et al., 2012; Bain et al., 2013; Becker et al., 2016; Schippers et al., 2016).

There are other additional functions of Ly6C monocytes (Figure 3). Studies have shown that CCL2, CCL3 and CCL11 may come from Ly6C^*hi*^ and play a role in recruiting innate immune effector cells in the gut (Waddell et al., 2011; Schulthess et al., 2012; Bain and Mowat, 2014). Ly6C^*hi*^ monocytes can also prevent immunopathology by inhibiting the production of TNF-α and ROI by local neutrophils (Grainger et al., 2013). Furthermore, intestinal macrophages participate in tissue repair and fibrosis (Figure 3; Karin and Clevers, 2016; Vannella and Wynn, 2017). Some with Crohn’s disease have symptoms of intestinal fibrostenosis, while others develop fibrosis complications several years later (Rieder et al., 2017). Another study has shown that in the colon of patients with Crohn’s disease with stenosis, the number of IL-36α^+^ macrophages in the intestine is increased (Scheibe et al., 2019). The direct effect of IL-36 on human mesenchymal cells leads to profibrosis transcription, which indicates that intestinal fibrosis in patients with IBD can be induced by the increase of IL 36α^+^ macrophages (Bettenworth and Rieder, 2017; Salvador et al., 2018; Scheibe et al., 2019). Other studies have reported that immature macrophages are always close to activated fibroblasts in the intestinal mucosa of patients with Crohn’s disease, and immature macrophages, as well as conventional dendritic cells 2, activate fibroblasts to induce intestinal inflammation by oncostatin M/OSMR signaling (West et al., 2017; Martin et al., 2019; Smillie et al., 2019). Some genes expressed by intestinal macrophages also affect the development of intestinal inflammation. For example, the gene ablation of GPBAR1, a G protein–coupled receptor that is highly expressed in macrophages, enhances the recruitment of classically activated macrophages in the colonic lamina propria and aggravates the severity of inflammation (Biagioli et al., 2017).

Macrophages also play a role in the activation of T cells (Figure 3). Some studies have shown that, in patients with Crohn’s disease, intestinal macrophages can induce the proliferation of naïve CD4^+^ T cells and the expression of integrin β-7 and CCR9, while other works deem mature macrophages from patients with ulcerative colitis unable to inhibit the proliferation of T cells (Barman et al., 2016). In addition, CD14^*hi*^ monocytes/macrophages in the IBD mucosa can produce IL-23 and express CD40 and CD80 to support the function of pathogenic T cells (Rugtveit et al., 1997; Carlsen et al., 2006; Kamada et al., 2008). Intestinal macrophages in patients with Crohn’s disease induce the Th1 and Th17 polarization of naïve CD4^+^ T cells, which seems to be caused by the accumulation of immature macrophages in the total macrophage population of patients with Crohn’s disease (Kamada et al., 2009; Ogino et al., 2013). In fact, it has been proved by previous studies that immature macrophages from patients with IBD mainly produce IL-1β to induce Th17 cells and pathological IFN-γ^+^ IL-17^+^ T cells, which come from autologous colon CD4^+^ T cells (Ramesh et al., 2014; Chapuy et al., 2019; Chapuy et al., 2020).

### The Role of Intestinal Macrophages in the Treatment of Inflammation and Diseases

There are many methods to treat IBD, and regulating macrophage activation is one of them. In fact, it has been considered an attractive treatment for IBD to increase the phenotype of anti-inflammatory M2 (Hidalgo-Garcia et al., 2018). Endoplasmic reticulum stress, which is involved in the regulation of IEC inflammatory injury, is common in IBD patients (Woehlbier and Hetz, 2011; Hosomi et al., 2015). Grp78 is a marker of endoplasmic reticulum stress, and its expression is increased in inflammatory IEC. However, after increasing the expression of IL-10, the expression of Grp78 decreases, and endoplasmic reticulum stress is inhibited (Shkoda et al., 2007). IL-10 inhibits the NF-κB RelA phosphorylation induced by TNF by regulating Grp78, the expression of pro-inflammatory cytokines is subsequently down-regulated, and the IEC barrier function is maintained. According to a previous study, the neutralization of IL-10/TGF-β or alternatively activated macrophages did not show resistance to colitis induced by DSS in mice infected with schistosome (Smith et al., 2007). Parasites inhibited colitis induced by DSS through a new colonic infiltrating macrophage population—i.e., the schistosome infection stimulates a new macrophage population that preferentially migrates to the colonic LP, where it can inhibit colitis (Smith et al., 2007). This finding highlights a variety of immunomodulatory macrophage activation states. It is worth noting that infliximab, a monoclonal antibody of anti-TNF-α, has been successfully used in the treatment of human IBD, and the regulatory macrophages CD68^+^CD206^+^ were induced in patients with IBD responsive to treatment (Vos et al., 2012; Danese et al., 2015). Some studies have proved that macrophages are significant for the treatment of IBD. For example, alternative activated macrophages can activate the Wnt signaling pathway, which is related to ulcerative colitis, and promote mucosal repair in IBD, while Yes-associated protein (YAP), a Hippo pathway molecule, can aggravate the occurrence of IBD by regulating macrophage polarization and the imbalance of intestinal flora homeostasis (Cosín-Roger et al., 2016; Zhou et al., 2019).

Macrophages play an important role in in the treatment of colitis. For example, it has been found that intracolonic administration of chromofungin can induce macrophages to enter alternatively activated macrophages (AAM), which reduce the deposition of colonic collagen and maintain the homeostasis of intestinal epithelial cells, thus protecting colitis induced by DSS (Eissa et al., 2017; Ding et al., 2019a). MicroRNAs (miRNAs), which are noncoding RNAs, are essential for many biological processes in fine tuning. In macrophages, miR-155 acts as a pro-inflammatory regulator by promoting M2 polarization or affecting NF-κB signal transduction (Vigorito et al., 2013; Zhang et al., 2016). Li et al. found the central role of alternative M2 skewing of miR-155 in colitis and suggested that macrophages might be the main target of treatment (Li et al., 2018). The Grb2-associated binding protein 2 (Gab2), which plays a role in regulating the activation of macrophages and T cells, and Grb2-associated binding protein 3 (Gab3), which is highly expressed in some immune cell types, redundantly regulate the activation of macrophages and CD8^+^ T cells to inhibit colitis (Uno et al., 2010; Bezman et al., 2012; Best et al., 2013; Festuccia et al., 2014; Kaneda et al., 2016; Wang et al., 2019; Ma et al., 2020b). Human catestatin (hCT), which has immunomodulatory properties, can reduce the severity of inflammatory recurrence by regulating M1 macrophages and releasing pro-inflammatory cytokines (Zhang et al., 2009; Rabbi et al., 2017). Triggering receptor expressed on myeloid cells-1 (TREM-1) is a pattern recognition receptor (PRR) of the surface immunoglobulin receptor superfamily and is expressed by activated macrophages. A study has found that when TREM-1 is deficient, the number of M1 macrophages, which produce IL-1β, in DSS-treated colons decreases, and the damage mediated by DSS can be alleviated by providing TREM-1 expressing macrophages to TREM-1 deficient mice (Yang et al., 2019). Other studies have found that vitamin D supplementation can also reduce the severity of Crohn’s disease, and its active form, 1,25-dihydroxyvitamin D (1,25D), can inhibit the secretion of pro-inflammatory cytokines by macrophages (Dionne et al., 2017). Moreover, 1,25 D is also very important for the regulation of bone homeostasis and various immune responses (Hewison, 2012).

In addition to inhibiting intestinal inflammation, macrophages also play a significant role in other diseases. For example, REG3γ is a secretory antimicrobial lectin and REG3γ-associated *Lactobacillus* can enlarge the macrophage pools in the intestinal lamina propria, spleen and adipose tissue. The anti-inflammatory macrophages induced by REG3γ-associated *Lactobacillus* in the lamina propria may migrate to the adipose tissue and participate in the resistance to high-fat-diet-mediated obesity, and adipose tissue homeostasis (Huang et al., 2017). Since the gastrointestinal tract contains many HIV target cells, it has become the main site of HIV infection. Some studies have shown that Toll-like receptor 3 activation of macrophages can produce a variety of intracellular HIV limiting factors and effectively inhibit HIV infection (Wang et al., 2013; Zhou et al., 2013). The supernatant of activated intestinal epithelial cells can induce macrophages to express several key HIV limiting factors, thus inhibiting the replication of HIV (Guo et al., 2018). Whether in mice or human, the cross-talk between liver and intestine is vital in the development of metabolic diseases (Zhang et al., 2010; Qin et al., 2014). For example, non-alcoholic fatty liver disease is usually accompanied by changes in the intestinal microflora and bacterial overgrowth these are related to increased intestinal permeability and pathological bacterial translocation, in which macrophages may also be involved (Bain and Mowat, 2014; Hundertmark et al., 2018). Macrophage inducible C-type lectin expressed on macrophages may contribute to the integrity of the intestinal barrier, but in the advanced stages of chronic liver disease, once the intestinal barrier leaks, it seems to cause inflammation and fibrosis (Schierwagen et al., 2020). Receptor-interacting protein (RIP)-3, a member of the serine threonine kinase family, is the central mediator of necrosis and is associated with many human diseases (Ramachandran et al., 2013; Roychowdhury et al., 2013; Linkermann and Green, 2014). It has been shown that the deficiency of RIP3 can inhibit macrophage accumulation and reduce inflammation in mice by inhibiting the TLR4–NF-kB pathway, and thus may be a potential therapeutic target for immune-mediated liver fibrosis (Wei et al., 2019).

Cytokine blockade has been used to suppress intestinal inflammation, but there are still some problems that should be considered, such as the prediction of the therapeutic effect and its prospect. Treating IBD by treating anti-tumor factors is an important breakthrough. However, many treatments have not achieved satisfactory results, and although some treatments are promising in animal models, they have not yet undergone rigorous clinical trials. Moreover, the deficiency of intestinal macrophages may increase the susceptibility to infection and inhibit the activity of tissue repair. Therefore, the potential risks associated with this immunotherapy require careful monitoring procedures. Other ways to improve intestinal homeostasis may consist of promoting the anti-inflammatory effects of macrophages. It is worth noting that, due to their high phagocytic capacity, intestinal macrophages can be promoted through “delivery systems” such as nanomaterials and biomaterials. Finally, the reprogramming of macrophages with metabolites may be a promising method to inhibit intestinal inflammation.

## Summary and Prospect

This paper reviews the origin, development, and function of macrophages and their role in intestinal inflammation and treatment. In the past few years, we have made significant progress in understanding the ontogeny and differentiation of intestinal macrophages. Advancements have been made in the recognition and regulation of tissue-specific phenotypes and functional environmental signals as well. Macrophages not only have the function of phagocytizing pathogens, but can also secrete a variety of cytokines under certain conditions and combine with different immune cells to participate in the occurrence, development, and persistence of IBD in different ways. At the same time, macrophages play a role in treating IBD, inhibiting colitis, maintaining adipose tissue homeostasis, and inhibiting HIV infection. In conclusion, macrophages are vital in gut homeostasis and immune defense. However, many aspects of intestinal macrophages still need to be explored. For example, the understanding of heterogeneity in the septum of intestinal macrophages needs to be more complete. An important feature of IBD is pro-inflammatory monocyte/macrophage accumulation. Therefore, it is very important to elucidate the exact character of the molecular factors that control the differentiation of monocyte/macrophage, the changes of these factors in the course of disease, the local regulation, and long-term effects. In addition, the study of the interaction between macrophages and other cells, intestinal microorganisms and metabolites will also contribute to the treatment of intestinal inflammation.

## Author Contributions

XH did the writing. SD did the writing—review and editing. HJ did the supervision. GL did the funding acquisition. All authors contributed to the article and approved the submitted version.

## Conflict of Interest

The authors declare that the research was conducted in the absence of any commercial or financial relationships that could be construed as a potential conflict of interest.
